# 

**Published:** 1947

**Authors:** 


### The Bristol Medico-Chirurgical Journal

A JOURNAL OF THE MEDICAL SCIENCES FOR THE

WEST OF ENGLAND AND SOUTH WALES

PUBLISHED UNDER THE AUSPICES OF
*THE BRISTOL MEDICO-CHIRURGICAL SOCIETY*

EDITOR
J. A. NIXON, C.M.G., M.D., F.R.C.P.
WITH WHOM ARE ASSOCIATED
A. RENDLE SHORT, M.D., B.S., B.Sc., F.R.C.S.
W. MELVILLE CAPPER, M.B., B.S., F.R.C.S.
G. E. F. SUTTON, M.C., M.D., B.S., M.R.C.P.
E. WATSON-WILLIAMS, M.C., Ch.M., F.R.C.S., Editorial Secretary

"Scire est nescire, nisi id me

Scire alius sciret"

*VOL. LXIV*

BRISTOL: J. W. ARROWSMITH LTD.

WI
BR323

### Contents of Vol. LXIV

Page

Back to Work. Presidential Address. By H. Chitty, M.S., F.R.C.S. .. 1

The Treatment of Subacute Bacterial Endocarditis with Penicillin. By C. Bruce Perry, M.D., Ch.B., F.R.C.P. .. .. .. .. .. 6

Resection of the Head of the Pancreas. By F. D. Murphy, O.B.E., F.R.C.S. 8

Upper Parietal Meningioma showing Foster Kennedy Syndrome. By Diana J. Kinloch Beck, M.B., B.S., F.R.C.S., F.R.C.S.E. .. .. 11

The Thirty-fifth Long Fox Memorial Lecture: Some Aspects of Aviation Medicine. By K. G. Bergin, M.A., M.D., A.F.R.Æ.S., *A.D.M.S., B.O.A.C.* 31

Penicillin in Diseases of the Ear, Nose and Throat. By E. Watson-Williams, *M.C.*, Ch.M. .. .. .. .. .. .. .. .. .. 42

Cardiac Anomalies Associated with Funnel Chest. By G. E. F. Sutton, *M.C.*, M.D., M.R.C.P. .. .. .. .. .. .. .. .. 45

War Injuries of the Face and Jaws. By G. M. FitzGibbon, M.D., F.R.C.S. .. 61

The Rhesus Factors—I: Serology. By Geoffrey H. Tovey, M.D. .. 68

The Rhesus Factors—II: Haemolytic Disease of the New-Born. By Beryl D. Corner, M.D., B.S., M.R.C.P. .. .. .. .. .. 74

Diagnosis of Disease in Infancy. By A. V. Neale, M.D., F.R.C.P. .. .. 93

Psychosomatic Dermatoses. By W. J. O'Donovan, M.D., O.B.E. .. 98

Poliomyelitis in Bristol, 1947. By James Macrae, M.D., F.R.F.P.S., D.P.H. 101

House of John Wright .. .. .. .. .. .. .. .. 106

Reviews of Books .. .. .. .. .. .. .. 13, 49, 81, 108

Editorial Notes .. .. .. .. .. .. .. .. 16, 51, 85

Non-Editorial Note .. .. .. .. .. .. .. .. 52

Obituary .. .. .. .. .. .. .. .. .. 54

Meetings of Societies .. .. .. .. .. .. .. 17, 55, 86

The Medical Library of the University of Bristol .. 18, 55, 86, 111

Publications Received .. .. .. .. .. .. 19, 57, 89, 113

Local Medical Notes .. .. .. .. .. .. 20, 58, 90, 114

432/70

### Index to Vol. LXIV

Adams, S. B.—Obituary, 54

Appointments, 20, 58, 90

Aviation Medicine, Some Aspects of.—K. G. Bergin, 31

Back to Work. Presidential Address.—H. Chitty, 1

Beck, D. J. K.—Upper Parietal Meningioma showing Foster Kennedy Syndrome, 11

Bergin, K. G.—Some Aspects of Aviation Medicine, 31

Book Reviews, 13, 49, 81, 108

Bristol, 1947, Poliomyelitis in.—J. Macrae, 101

ardiac Anomalies Associated with Funnel Chest.—G. E. F. Sutton, 45

hitty, H.—Back to Work : Presidential Address, 1

orner, B. D.—Rhesus Factors : Haemolytic Disease of the New-Born, 74

ermatoses, Psychosomatic.—W. J. O'Donovan, 98

iagnosis of Disease in Infancy.—A. V. Neale, 93

isease in Infancy, Diagnosis of.—A. V. Neale, 93

ar, Nose and Throat, Penicillin in Diseases of the.—E. Watson-Williams, 42

litorial Notes :

Nursing, 16

Regional Hospital Board, 51

German Educational Reconstruction, 52

The Future of the *Journal*, 85

ndocarditis, Subacute Bacterial, Treatment of, with Penicillin.—C. B. Perry, 6

ce and Jaws, War Injuries of.—G. M. FitzGibbon, 61

zGibbon, G. M.—War Injuries of the Face and Jaws, 61

ster Kennedy Syndrome in Upper Parietal Meningioma.—D. J. K. Beck, 11

Funnel Chest, Cardiac Anomalies Associated with.—G. E. F. Sutton, 45

German Educational Reconstruction : Editorial Note, 52

House of John Wright, 106

Jaws, War Injuries of Face and.—G. M. FitzGibbon, 61

John Wright, House of, 106

*Journal*, The Future of the.—Editorial Note, 85

Library Notes, 18, 55, 86, 111

Long Fox Memorial Lecture, XXXV.—K. G. Bergin, Some Aspects of Aviation Medicine, 31

Macrae, J.—Poliomyelitis in Bristol, 1947, 101

Meningioma, Upper Parietal, showing Foster Kennedy Syndrome.—D. J. K. Beck, 11

Murphy, F. D.—Resection of the Head of the Pancreas, 8

Neale, A. V.—Diagnosis of Disease in Infancy, 93

New-Born, Haemolytic Disease of the.—B. D. Corner, 74

Nixon, J. A.—Twenty-one Years Editor, 52

Nursing.—Editorial Note, 16

Obituary.—S. B. Adams, 54

O'Donovan, W. J.—Psychosomatic Dermatoses, 98

Pancreas, Resection of the Head of the.—F. D. Murphy, 8

Penicillin in Diseases of the Ear, Nose and Throat—E. Watson-Williams, 42

Penicillin in Subacute Bacterial Endocarditis.—C. Bruce Perry, 6

Perry, C. B.—Treatment of Subacute Bacterial Endocarditis with Penicillin, 6

Poliomyelitis in Bristol, 1947.—J. Macrea, 101

Presidential Address: Back to Work.—H. Chitty, 1

Psychosomatic Dermatoses. — W. J. O'Donovan, 98

Regional Hospital Board.—Editorial Note, 51

Resection of the Head of the Pancreas.—F. D. Murphy, 8

Reviews of Books, 13, 49, 81, 108

Rhesus Factors:
Serology.—G. H. Tovey, 68
Haemolytic Diseases of the New-Born.—B. D. Corner, 74

Some Aspects of Aviation Medicine·
K. G. Bergin, 31

Sutton, G. E. F.—Cardiac Anomali
Associated with Funnel Chest, 45

Tovey, G. H.—Rhesus Factors: Ser logy, 68

Upper Parietal Meningioma showi

Foster Kennedy Syndrome.—D.

K. Beck, 11

War Injuries of the Face and Jaws·

G. M. FitzGibbon, 61

Watson-Williams, E. — Penicillin
Diseases of the Ear, Nose a
Throat, 42

### The Medical Library of the University of Bristol

WITH WHICH IS INCORPORATED

**The Library of the Bristol Medico-Chirurgical Society.**

---

*The following donations have been received since the publication of the list in the last issue.*

| | |
|---|---|
| H. G. Garde, Esq. (1) | 1 volume. |
| Professor T. F. Hewer (2) | 14 volumes. |
| L'Institut Pasteur d'Alger (3) | 1 volume. |
| Middlesex Hospital Medical School (4) | 1 volume. |
| Royal College of Physicians (5) | 1 volume. |
| Royal College of Surgeons (6) | 1 volume. |
| Dr. G. de M. Rudolf (7) | 1 volume. |
| J. E. Shaw Bequest (8) | 2 volumes. |
| Dr. G. E. F. Sutton (9) | 1 volume. |
| Dr. W. V. Wood (10) | 1 volume. |
| Dr. Arthur Wright (11) | 6 volumes. |

Unbound periodicals have also been received from Professor T. F. Hewer, Dr. G. K. McGowan, Professor Milnes Walker, and Messrs. John Wright & Sons Ltd.

### THE ONE HUNDRED AND EIGHTY-THIRD LIST OF BOOKS.

The figures in round brackets refer to the figures after the names of the donors. The books to which no such figures have been attached have either been bought from the Library Fund or received through the *Journal.*

| | | | |
|---|---|---|---|
| **Best, C. H., and Taylor, N. B.** | *Physiological Basis of Medical Practice* | 4th Ed. | 1945 |
| **Boyd, W.** | *Introduction to Medical Science* | 3rd Ed. | 1945 |
| **Cope, V. Z.** | *Early Diagnosis of the Acute Abdomen* | 9th Ed. | 1946 |
| **Cullen, S. C.** | *Anæsthesia in General Practice* | | 1946 |
| **Cunningham, E. R.** | *Classification for Medical Literature* | 3rd Ed. | 1946 |
| **Dyke, S. C. (Ed.)** | *Recent Advances in Clinical Pathology* | | 1947 |
| **Findlay, A.** | *Physical Chemistry for Students of Medicine* | (10) | 1927 |
| **Fulton, J. F., and Stanton, M. E.** | *Centennial of Surgical Anæsthesia* | | 1946 |
| **Garde, H. G.** | *A Contribution to the War Against Cancer?* | (1) | 1947 |
| **Geschickter, C. F., and Copeland, M. M.** | *Tumors of Bone* | | 1936 |
| **Ghosh, B. N.** | *Treatise on Hygiene and Public Health* | 11th Ed. | 1945 |
| **Harrow, B.** | *Textbook of Biochemistry* | 4th Ed. | 1946 |
| **Kershaw, J. D.** | *An Approach to Social Medicine* | | 1946 |
| **Lennox, W. G.** | *Science and Seizures* | 2nd Ed. | 1946 |

| | | |
|---|---|---|
| **Malloch, A.** .. .. .. .. | *Medical Interchange between the British Isles and America before* 1801 .. .. .. (5) | 1946 |
| **Morton, W. T. G.** .. .. | *Memoir to the Academy of Sciences at Paris on a New Use of Sulphuric Ether*, 1847 .. .. | 1946 |
| *New and Non-Official Remedies*, 1946 .. .. .. .. | | 1946 |
| **Percival, G. H., and others** .. | *Atlas of Histopathology of the Skin* .. .. .. | 1947 |
| **Perkin, F. M.** .. .. .. | *Qualitative Chemical Analysis* .. .. 4th Ed. | 1928 |
| **Pillmore, G, U.** .. .. .. | *Clinical Radiology*, 2 Vols. .. .. .. (8) | 1946 |
| **Powell, Sir Allan** .. .. | *Metropolitan Asylums Board and Its Work*, 1867–1930 .. .. .. .. .. .. .. (7) | 1930 |
| **Price, H. P.** .. .. .. .. | *Short Handbook of Practical Anæsthetics* .. .. | 1946 |
| **Rolleston, Sir H. (Ed.)** .. | *British Encyclopædia of Medical Practice: Surveys and Abstracts*, 1939–40; *Medical Progress*, 1941–45 .. .. .. .. (11) | 1939–45 |
| **Ross, J. A.** .. .. .. .. | *Handbook of Radiography* .. .. .. 2dn Ed. | 1946 |
| **Ross, J. M.** .. .. .. .. | *Post-mortem Appearances* .. .. .. 4th Ed. | 1944 |
| **Sergent, E., and others** .. | *Etudes sur les Piroplasmoses Bovines* .. .. (3) | 1945 |
| **Singer, C., and Rabin, C.** .. | *Prelude to Modern Science* .. .. .. .. | 1946 |
| **Short, A. R.** .. .. .. | *Causation of Appendicitis* .. .. .. .. .. .. | 1946 |
| **Stieglitz, E. J.** .. .. .. | *A Future for Preventive Medicine* .. .. .. | 1945 |
| **Sutton, G. E. F.** .. .. .. | *Aids to Medical Diagnosis* .. 6th Ed. (9) | 1946 |
| **Walker, K., and Strauss, E. B.** | *Sexual Disorders in the Male* .. 2nd Ed. | 1944 |
| **Walshe, F. M. R.** .. .. | *Diseases of the Nervous System* .. .. 5th Ed. | 1947 |
| **Weber, F. P.** .. .. .. | *More Thoughts of a Doctor*, I. .. .. .. .. .. | 1938 |
| **Weber, F. P.** .. .. .. | *More Thoughts and Comments of a Doctor*, II.–VIII. .. .. .. .. .. .. .. .. .. | 1946 |

TRANSACTIONS, REPORTS, JOURNALS, ETC.

| | | |
|---|---|---|
| Annual Report of the Smithsonian Institution, 1945 .. .. .. .. .. .. | | 1946 |
| Calendar of the Royal College of Surgeons of England, 1946 .. .. .. | (6) | 1946 |
| Collected Papers of the Middlesex Hospital Medical School, 1944–46 .. | (4) | 1947 |
| Medical Annual, 1946 .. .. .. .. .. .. .. .. .. .. .. .. .. | | 1946 |
| Quarterly Cumulative Index Medicus. Vol. 39, Jan.–June, 1946 .. .. .. | | 1947 |
| Transactions of the Royal Society of Tropical Medicine and Hygiene. Vol. 27 (1934)—Vol. 40 (1946) .. .. .. .. .. .. .. .. .. .. | (2) | |
| Year-book of Eye, Ear, Nose and Throat, 1946 .. .. .. .. .. .. .. | | 1946 |
| Year-book of Radiology, 1946 .. .. .. .. .. .. .. .. .. .. .. .. | | 1946 |

### Publications Received

From OXFORD UNIVERSITY PRESS:

*Diseases of the Heart and Circulation.* By ALBERT A. F. PEEL. 1947. Price 35s.

FROM MEDICAL SCIENCE PUBLISHERS, WASHINGTON, D.C.:

*Case Studies in the Psychopathology of Crime.* Vol. 2. By BEN KARPMAN.

From MESSRS. JOHN WRIGHT & SONS LTD.:

*Medical Annual,* 1946. Edited by Sir HENRY TIDY and A. RENDLE SHORT. 1946. Price 25s.

*Pye's Surgical Handicraft.* Edited by HAMILTON BAILEY. Fifteenth Edition. 1947. Price 25s.

*British Journal of Surgery.* Vol. 34. No. 135. January, 1947. Price 12s. 6d.

### Local Medical Notes

BRISTOL ROYAL HOSPITAL.

The following appointments have been made : *Honorary Physicians.*—A. M. G. Campbell, D.M., M.R.C.P., and D. H. Davies, M.B., B.Ch., M.R.C.P. *Honorary Psychiatric Physicians.*—R. E. Hemphill, M.D., D.P.M., and R. F. Barbour, M.B., F.R.C.P., D.P.M. *Honorary Surgeons.*—W. Melville Capper, M.B., B.S., F.R.C.S., and J. A. Pocock, F.R.C.S. *Honorary Orthopædic Surgeon.*—A. L. Eyre-Brook, M.B., M.S., F.R.C.S. *Honorary Gynæcologist.*—Miss Mabel Potter, M.D., Ch.B., F.R.C.O.G.

EXAMINATION RESULTS.

Students of the University have recently passed the following examinations :—

**University of Bristol.**—M.B., Ch.B.—*Second Examination* (*Section I*) : R. J. Ancill (*a*), P. J. D. Benjafield, Monica B. Bishop, H. J. Bland, K. A. Boulton, Margaret V. Brown, Margaret H. Buston, C. E. Dickson, Dorothy R. Douglas, E. R. Duchesne, W. A. W. Dutton, Doreen A. Ellis (*a*), B. R. W. Evans, M. A. Feldman, Z. Fluss, R. F. Harvey, R. Hill, E. A. W. Houghton, G. A. K. M'C. Jahans, J. A. Jefferies, K. R. Kidd, Helena B. M. Knottova, H. I. Leigh (*a*), Jean M. Liell, S. J. Lloyd, H. Maxwell, J. Oates, Barbara M. Philpott, J. F. Rowland, Hella R. Sadler, Rachel D. R. Sasieni, M. Sheldrick, R. E. D. Simpson, L. P. Spence, D. T. I. Stuttaford, Marianne A. Taylor, Ginette M. Tebbutt, P. A. Titterton, L. A. D. Tovey, M. D. Turner, Joan Walker.

(*a*) With Distinction in Anatomy.

### Bristol Medico-Chirurgical Society

### President.

H. CHITTY, M.S. Lond., F.R.C.S.

### President-Elect.

T. MILLING, M.B., Ch.B.

### Committee.

*Ex-Officio* { J. A. NIXON, *C.M.G.*, B.A., M.D. Cantab. (Editor of *Journal*).

(Vacant), (Hon. Librarian).

*Elected.*

R. F. BARBOUR, M.A., F.R.C.P., D.P.M.
F. H. BODMAN, M.D.
R. V. COOKE, Ch.M., F.R.C.S.
BERYL D. CORNER, M.D.
H. E. HARRIS, M.B., Ch.B., F.R.C.S.
G. D. KERSLEY, M.A., M.D., F.R.C.P.
G. L. ORMEROD, M.A., M.B., B.Chir.
A. PALIN, B.M., B.Ch.
H. K. V. SOLTAU, M.D.

### Honorary Secretary.

A. L. EYRE-BROOK, M.S.

### Honorary Treasurer.

FREDERICK SUTTON, M.C., M.D.

### University Library Sub-Committee.

A. R. SHORT, B.Sc., M.D. Lond., F.R.C.S.
C. BRUCE PERRY, M.D. Brist., F.R.C.P.
H. J. DREW SMYTHE, *M.C.*, M.S., M.D. Lond., F.R.C.S., F.R.C.O.G.

Medical Practitioners wishing to join the Society are requested to communicate with the Hon. Secretary, Mr. A. L. Eyre-Brook, Litfield House, Clifton Down, Bristol 8. The Annual Subscription is £1 1s., payable to Hon. Treasurer.

*Extracts from Journal By-laws.*

4.—The *Journal* shall be delivered free to Subscribers and also to Members of the Society whose subscriptions are not in arrear.

6.—The Annual Subscription to the *Journal* is 16/–, payable to Hon. Treasurer.

Communications intended for insertion in the *Journal* should be sent to the Editor, Dr. J. A. Nixon, 7 Lansdown Place, Clifton, Bristol.

Books for Review and Exchange Journals should be sent to the Assistant Editor, *Bristol Medico-Chirurgical Journal,* Medical Library, The University, Bristol. Reviews should be returned with the books to Mr. W. M. Capper, Litfield House, Clifton Down, Bristol 8.

Communications referring to the delivery of the *Journal* should be sent to the Hon. Treasurer, Dr. Frederick Sutton, 1 Pembroke Road, Bristol 8.

Volumes of the *Journal* can be bound upon application to J. W. Arrowsmith Ltd., Quay Street, Bristol, price on request. The inclusive Index for Volumes I—L may be obtained, price 3/– (postage extra).

### Bristol Medico-Chirurgical Society

### PAST PRESIDENTS

1874—75. FREDERICK BRITTAN, M.D., B.S. Dub.
1875—76. ROBERT WILLIAM COE.
1876—77. SAMUEL MARTYN, M.D. Ed.
1877—78. AUGUSTIN PRICHARD, M.D. Berlin.
1878—79. HENRY EDWARD FRIPP, M.D. Würzburg.
1879—80. WILLIAM MICHELL CLARKE.
1880—81. JOSEPH GRIFFITHS SWAYNE, M.D. Lond.
1881—82. EDWARD LONG FOX, M.D. Oxon.
1882—83. JAMES GEORGE DAVEY, M.D. St. And.
1883—84. WILLIAM JOHNSTONE FYFFE, M.D., B.A. Dub.
1884—85. GEORGE FORSTER BURDER, M.D. Aberd.
1885—86. WILLIAM HENRY SPENCER, M.D., M.A. Cantab.
1886—87. FRANCIS POOLE LANSDOWN.
1887—88. LEMUEL MATTHEWS GRIFFITHS.
1888—89. ROBERT SHINGLETON SMITH, M.D., B.Sc. Lond.
1889—90. NELSON CONGREVE DOBSON, Ch.M. Brist.
1890—91. SAMUEL HENRY SWAYNE.
1891—92. FRANCIS RICHARDSON CROSS, M.B. Lond., LL.D. Brist.
1892—93. EDWARD MARKHAM SKERRITT, M.D., B.S., B.A. Lond.
1893—94. JAMES GREIG SMITH, M.B., C.M., M.A. Aberd., F.R.S.E.
1894—95. ALFRED JAMES HARRISON, M.B. Lond.
1895—96. ARTHUR WILLIAM PRICHARD.
1896—97. ALFRED EDWARD AUST LAWRENCE, M.D., C.M. Aberd.
1897—98. JOHN EDWARD SHAW, M.B., C.M. Ed.
1898—99. ROBERT ROXBURGH, M.B. Ed.
1899—00. WILLIAM HENRY HARSANT.
1900—01. DAVID SAMUEL DAVIES, M.D. Lond.
1901—02. BARCLAY JOSIAH BARON, M.B., C.M. Ed.
1902—03. GEORGE MUNRO SMITH, M.D. Brist.
1903—04. JAMES PAUL BUSH, C.M.G., C.B.E., Ch.M. Brist.
1904—05. REGINALD EAGER, M.D. Lond.
1905—06. JOHN DACRE.
1906—07. JAMES TAYLOR.
1907—08. HENRY WALDO, M.D., C.M. Aberd.
1908—09. JOHN MICHELL CLARKE, M.D., M.A. Cantab.
1909—10. JAMES SWAIN, C.B., C.B.E., M.D., M.S. Lond.
1910—11. BERTRAM MITFORD HERON ROGERS, M.D., B.Ch., B.A. Oxon.
1911—12. CHARLES ALEXANDER MORTON.
1912—13. WALTER CARLESS SWAYNE, M.D., B.S. Lond. and Brist.
1913—14. PATRICK WATSON-WILLIAMS, M.D. Lond., M.D. (Hon. Causa) Brist.
1914—15. WILLIAM HARRY CHRISTOPHER NEWNHAM, M.A., M.B. Cantab.
1915—16. GEORGE PARKER, M.A., M.D. Cantab., LL.D. Brist.
1916—17. FRANCIS HENRY EDGEWORTH, M.A., M.D. Cantab., D.Sc. Lond.
1917—18. FREDERICK LACE.
1918—19. ROBERT GUTHRIE POOLE LANSDOWN, M.D., B.S. Durh.
1919—20. I. WALKER HALL, M.D., Ch.B. Vict.
1920—21. LEOPOLD ERNEST VALENTINE EVERY-CLAYTON, M.D. Lond.
1921—22. CYRIL HUTCHINSON WALKER, B.A., M.B. Cantab.
1922—23. JOHN ALEXANDER NIXON, C.M.G., B.A., M.D. Cantab.
1923—24. JOHN LACY FIRTH, M.D., M.S. Lond.
1924—25. JOHN ODERY SYMES, M.D. Lond.
1925—26. THOMAS CARWARDINE, M.S. Lond.
1926—27. ARTHUR LAUNCELOT FLEMMING, M.B., Ch.B. Brist.
1927—28. WALTER KENNETH WILLS, M.A., M.B., B.Ch. Cantab.
1928—29. HENRY LAWRENCE ORMEROD, M.D., B.Ch. R.U.I.
1929—30. CHARLES FERRIER WALTERS.
1930—31. DAVID CHARLES RAYNER, Ch.M. Brist.
1931—32. JOHN ROGER CHARLES, M.A., M.D. Cantab.
1932—33. ERNEST WILLIAM HEY GROVES, M.D., M.S., D.Sc., B.Sc. Lond.
1933—34. HERBERT ELWIN HARRIS, B.A., M.B. Cantab.
1934—35. A. RENDLE SHORT, B.Sc., M.D. Lond.
1935—36. ARTHUR ERNEST ILES, O.B.E., M.B., B.S. Lond.
1936—37. RICHARD CHRISTOPHER CLARKE, O.B.E, M.B. Lond.
1937—38. CLIFFORD A. MOORE, M.B., M.S. Lond.
1938—40. LEONARD AUGUSTUS MOORE, M.B., Ch.B. Brist.
1940—41. CHARLES CORFIELD, M.D. Durh., Barr.-at-Law.
1945—46. H. H. CARLETON, M.A., M.D. Oxon.
1946—47. H. CHITTY, M.S. Lond.

1947.

### LIST OF SUBSCRIBERS

TO THE

### Bristol Medico-Chirurgical Journal.

### HONORARY MEMBER OF THE SOCIETY.

| | |
|---|---|
| Swain, James, *C.B.*, *C.B.E.*, M.D., M.S. Lond. .. .. .. | Wyndham House, Easton-in-Gordano. |

### SUBSCRIBERS.

*Those marked * are Members of the Society.*

| | |
|---|---|
| *Adams, A. W., M.S. Lond. .. .. .. .. | Elton House, Clifton, Bristol 8. |
| Adams, J. B. .. .. .. .. .. .. .. .. .. | Kent Lodge, Eastbourne. |
| *Aldwinckle, Helen M., M.B., Ch.B. Brist. .. | 23 Rockland Road, Downend, Bristol. |
| *Alexander, A. J. P., M.D. Belf. .. .. .. | Winsley Sanatorium, near Bath. |
| *Alexander, D. A., M.B., Ch.B. Vict. .. .. | 112 Pembroke Road, Clifton, Bristol 8. |
| Alexander, G. L., B.A., M.B., B.Ch. Cantab. | West African Medical Service, Laura, via Accra, Gold Coast. |
| *Alford, A. F., M.B., Ch.B. Brist. .. .. | Ministry of Education, Belgrave Square, London, S.W.1. |
| Ashton, L. P., M.B., Ch.B. Brist. .. .. .. | Kapsowar, P.O. Eldoret, Kenya, East Africa. |
| *Baer, P., M.D. Milan .. .. .. .. .. .. | 10 Redland Park, Bristol 6. |
| *Bain, W., M.D. Glasg. .. .. .. .. .. .. | 1 Greville Road, Southville, Bristol 3. |
| *Baker, Lily A., M.D., B.Ch., B.A.O., R.U.I. | 23 Pembroke Road, Clifton, Bristol 8. |
| Ball, Mrs. J. J. .. .. .. .. .. .. .. .. | St. Leonard's Vicarage, Redfield, Bristol 5. |
| *Barbour, R. F., M.A. Camb., M.B., Ch.B. Edin. | 13 Old Sneed Road, Stoke Bishop, Bristol. |
| *Barnfield, R. H., M.R.C.S., L.R.C.P. .. | 72 Pembroke Raad, Clifton, Bristol 8. |
| *Baxter, Ursula, M.B., Ch.B. Brist. .. .. | 167 Wells Road, Knowle, Bristol 4. |
| *Baxter, V. T., M.B., Ch.B. Brist. .. .. .. | 167 Wells Road, Knowle, Bristol 4. |
| *Beatson, D. H., M.B., Ch.B. Brist. .. .. | 8 Richmond Park Road, Clifton, Bristol 8 |
| *Beck, Miss Diana J. Kinloch, M.B., B.S. Lond. | 8 Goldney Ave., Clifton, Bristol 8. |
| *Bell, R. J. Irving .. .. .. .. .. .. .. | 5a Oakfield Road, Clifton, Bristol 8. |
| *Belsey, R., M.S. Lond. .. .. .. .. .. .. | Frenchay Hospital, near Bristol. |
| *Bergin, F. G. .. .. .. .. .. .. .. .. .. | 31 Oakfield Road, Clifton, Bristol 8. |
| *Bergin, Kenneth, M.A., M.D. Cantab. .. | 31 Oakfield Road, Clifton, Bristol 8. |
| *Berry, R. J. A., Prof., M.D., C.M. Edin. .. | 3c All Saints' Road, Clifton, Bristol 8. |
| *Birrell, J. A., M.D., B.S. Lond. .. .. .. | 1 Victoria Square, Bristol 8. |
| *Bishop, G. W. R., *O.B.E.*, M.B., Ch.B. Brist. | Bloomfield House, High Street, Shepton Mallet. |
| *Black, W. R., M.B., Ch.B. Edin. .. .. .. | Southside, Rockleaze, Bristol 9. |
| *Bodenham, D. C., M.B., Ch.B. Brist. .. .. | 22 Royal York Crescent, Clifton, Bristol 8. |
| *Bodman, F. H., M.D., Ch.B. Brist. .. .. .. | 2 Redland Court Road, Bristol 6. |
| *Bolt, David, M.B., Ch.B. Brist. .. .. .. | 33 Knowle Road, Bristol 4. |
| Bolt, R. F. .. .. .. .. .. .. .. .. .. | 4 Gordon Mansions, Torrington Place, London, W.C.1. |
| *Boulton, B. J., M.B., Ch.B. Brist. .. .. | Ham Green Hospital, Pill, near Bristol. |
| *Bowles, Elizabeth M., M.B., B.S. Lond. .. | School Farm, Chew Stoke. |
| *Bown, Naomi J., M.B., Ch.B. Brist. .. .. | The Corner, Nailsea, Somerset. |
| *Boyd, R. G., M.B., Ch.B. Brist. .. .. .. | 44 Elm Lane, Bristol 6. |
| *Bradbeer, E. G., M.B., Ch.B. Brist. .. .. | 7 Avon Grove, Sneyd Park, Bristol 9. |
| *Brasher, C. W. J., M.D. Brist. .. .. .. | 17 Royal York Crescent, Clifton, Bristol 8. |
| *Brennen, R. G., M.B., B.Ch., B.A.O. Belf. .. | 19 Beaufort Road, Bristol 8. |
| *Brinckman, Winifred P., M.B., Ch.B. Brist. | 2 Clifton Park, Bristol 8. |
| *Britton, R. B., M.B., B.S. Lond. .. .. .. | 33a Whiteladies Road, Clifton, Bristol 8. |

| | |
|---|---|
| *BROCKLEHURST, R. J., M.A., D.M. Oxon. .. | The University, Bristol 8. |
| BROWN, J. E., M.B., Ch.B. Brist. .. .. .. .. | Coulthurst Villa, Brives Road, Maraval, Trinidad, B.W.I. |
| BRYAN, C. .. .. .. .. .. .. .. .. .. .. .. | 44 Nevil Road, Bishopston, Bristol 7. |
| *BRYANT, EUNICE .. .. .. .. .. .. .. .. .. | 167 Redland Road, Bristol 6. |
| *BURGES, R. .. .. .. .. .. .. .. .. .. .. | Druid's Mead, Stoke Bishop, Bristol 9. |
| *BUSH, G. B., M.B., Ch.B. Brist. .. .. .. | Litfield House, Clifton, Bristol 8. |
| *CAIRNS, F. J... .. .. .. .. .. .. .. .. .. | Devon House, Whitehall Road, Bristol 5. |
| CAMERON, MARGARET, M.B., Ch.B. Glasg. .. | 1 Dean Lane, Bristol 3. |
| *CAMPBELL, A. M. G., M..D Oxf. .. .. .. .. | 62 Pembroke Road, Clifton, Bristol 8. |
| *CAPPER, W. M., M.B., B.S. Lond. .. .. .. .. | 28 Downs Park West, Bristol 6. |
| *CAREY, R. S., *O.B.E.*, B.A. Cantab. .. .. | 502 Bath Road, Brislington, Bristol 4. |
| *CARLETON, H. H., M.A., M.D., B.Ch. Oxon. | 1 Lansdown Road, Clifton, Bristol 8. |
| *CARMICHAEL, T. G. .. .. .. .. .. .. .. .. | 140 Wick Road, Bristol 4. |
| *CASSON, A. H. .. .. .. .. .. .. .. .. .. | Hill House, Chipping Sodbury. |
| *CASSON, E., M.D., Ch.B. Brist... .. .. .. .. | Mount Pleasant, Clevedon. |
| *CHAMBERS, E. R... .. .. .. .. .. .. .. .. | 9 Clifton Park, Clifton, Bristol 8. |
| *CHARLES, J. R., M.A., M.D. Cantab. .. .. | Litfield House, Clifton Down, Bristol 8. |
| *CHITTY, H., M.S. Lond. .. .. .. .. .. .. | Naish House, Clapton-in-Gordano. |
| *CHRISTOFFERSON, P. E., M.B., Ch.B. Brist. .. | 14 Oakfield Road, Clifton, Bristol 8. |
| *CLAREMONT, L. E., M.D.S. Brist. .. .. .. | 5 Rodney Place, Clifton, Bristol 8. |
| *CLARK, DENNIS O., M.B., B.S. Lond. .. .. | 168 Milton Road, Weston-super-Mare. |
| *CLARKE, R. C., *O.B.E.*, M.B., Ch.B. Brist. .. | Harley Lodge, Clifton Park, Bristol 8. |
| *COCHRANE, J. A. .. .. .. .. .. .. .. .. | 11 Bryant's Hill, St. George, Bristol 5. |
| *COLLINGWOOD, F. C., M.B., Ch.B. Brist. .. | 106 Foxley Lane, Purley, Surrey. |
| *COOK, ELIZABETH M., M.D., Lond. .. .. | 15 Queen's Court, Clifton, Bristol 8. |
| *COOKE, R. V., Ch.M. Brist. .. .. .. .. | Litfield House, Bristol 8. |
| *COOKSON, R. G. .. .. .. .. .. .. .. .. .. | Clifton College, Bristol 8. |
| COOMBS, N. C. .. .. .. .. .. .. .. .. .. | 55 Galgate, Barnard Castle, Co. Durham. |
| *CORFIELD, C., M.D. Durh. .. .. .. .. .. .. | 5 Kensington Place, Clifton, Bristol 8. |
| *CORMACK, J. M., M.B., Ch.B. Glasg. .. .. | 1 Rodway Hill, Mangotsfield, Bristol. |
| *CORNER, BERYL, M.D., B.S. Lond. .. .. .. | 3 Elton House, Rodney Place, Bristol 8. |
| *COSSENS, K. M., B.Chir. Camb... .. .. .. | 52 Cotham Hill, Cotham, Bristol 6. |
| *COSSHAM, D. G., M.B., Ch.B. Brist. .. .. | Kingsleigh, Cirencester. |
| *COSSHAM, W. L., M.B., Ch.B. Brist. .. .. | 3 Claremont Road, Bishopston, Bristol 7. |
| *COURTNEY, R. M., M.B., Ch.B. Glasg. .. .. | Manor House, Southmead Road, Bristol |
| *COXON, BEATRICE .. .. .. .. .. .. .. .. | 16 Richmond Hill, Clifton, Bristol 8. |
| *CRAIG, ANNE A., M.D., B.S. Lond. .. .. .. | 35 Queen's Court, Clifton, Bristol 8. |
| *CRAIG, N. SMITH, M.B., Ch.B. Edin. .. .. | 7 Miles Road, Clifton, Bristol 8. |
| *CROOK, B. A., M.B., Ch.B. Brist. .. .. .. | The Laurels, Timsbury, near Bath. |
| *CROOT, H. J., M.B., B.S. Lond... .. .. .. | Dept. of Surgery, Royal Infirmary, Bristol 2. |
| *CROSSMAN, F. W., M.B. Durh. .. .. .. .. | White's Hill, Hambrook, near Bristol. |
| DAMMREL (Mrs.), M.B., Ch.B. .. .. .. .. .. | Henley Lodge, Yatton, Som. |
| *DATTA, S. S., M.D. Brist. .. .. .. .. .. .. | Snowdon House, Fishponds, Bristol. |
| *DAVIES, D. H., M.B. .. .. .. .. .. .. .. | Charlton, Leigh Woods, Bristol. |
| *DAVIES, E. D. D. .. .. .. .. .. .. .. | Battledown Court, Cheltenham, Glos. |
| *DAVIES, J. N. P., M.B., Ch.B. Brist. .. .. | Path. Department, Mulago Hospital, P.O. 351, Kampala, Uganda. |
| *DELANEY, N., M.B., Ch.B. Manch. .. .. .. | The Meade, Bishopsworth, Somerset. |
| *DISNEY, M., M.D. Lond. .. .. .. .. .. .. | 2 Clifton Park, Bristol 8. |
| *DIX, C. .. .. .. .. .. .. .. .. .. .. .. | 14 Mortimer Road, Clifton, Bristol 8 |
| *DIXON, MARY, M.B., Ch.B. Brist. .. .. .. | 11 Pembroke Road, Bristol 8. |
| *DIXON, T. B., *C.B.E.*, V.D. .. .. .. .. .. | 11 Pembroke Road, Bristol 8. |
| *DODD, GRANTHAM, M.B., B.S. Durh. .. .. | 42 Woodstock Road, Bristol 6. |
| *DOWLING, A.A., M.B., Ch.B. Brist. .. .. .. | 13 Dowry Square, Bristol 8. |
| *DUERDEN, J. R., M.B., Ch.B. Brist. .. .. | 4 Cecil Road, Bristol 8. |
| *DUNSTER, MARJORIE, M.D. Lond., Ch.M. Brist. | Bristol General Hospital (St. Monica's). |
| *DUTTON, HILDA R. .. .. .. .. .. .. .. | 405 Stapleton Road, Bristol 5. |
| *EGLINTON, J. H. C. .. .. .. .. .. .. .. | Street, Somerset. |
| *ELLIOTT, W. H. A., M.B., B.S. Lond. .. .. | 29 Old Sneed Avenue, Stoke Bishop, Bristol 9. |

| | |
|---|---|
| *ELLIOTT, Lt.-Com. JOHN, M.B., Ch.B. Brist. .. | 29 Old Sneed Avenue, Bristol 9. |
| *ELLIOTT, SQ.-LEADER W. H. GREVILLE, M.B., Ch.B. Brist. .. .. .. .. .. .. .. .. .. | 100 Redland Road, Bristol 6. |
| *ELLIS, W. T. .. .. .. .. .. .. .. .. .. .. | Tregenna House, Shirehampton. |
| *EMERY, J. L., M.D. Brist. .. .. .. .. .. .. | Children's Hospital, Bristol 2. |
| *EVANS, CLIFFORD, *O.B.E.*, M.B., B.Ch. Camb. | Litfield House, Clifton Down, Bristol 8. |
| *EVANS, G. M., M.B., Ch.B. Brist. .. .. .. .. | 117 Ashley Road, Bristol. |
| *EYRE-BROOK, A. L., M.B., M.S. Lond. .. .. | Litfield House, Bristol 8. |
| *EYRE-BROOK, E. M., M.B., Ch.B. Brist. .. .. | Litfield House, Bristol 8. |
| *FAIRMAN, H. D., M.B., B.S. Lond. .. .. .. | 50 Reedley Road, Bristol 9. |
| *FAIRWEATHER, D. S., M.A., M.D. Edin. .. .. | Purdown House, Stapleton, Bristol. |
| *FAYLE, B. W. D., B.A., M.B., B.Ch., B.A.O. Dub. | Galen House, Dursley, Glos. |
| *FELLS, G. R., M.B., Ch.B. Brist. .. .. .. .. | 19 Southmead Road, Bristol. |
| *FELLS, R. R., M.B., B.S. Lond. .. .. .. | 17 Mortimer Road, Clifton, Bristol 8. |
| *FENELEY, G. L., M.B., Ch.B. Brist. .. .. | Englewood, Falcondale Road, Westbury-on-Trym. |
| *FINZEL, H. F. M., M.D. Brist. .. .. .. .. .. | The Beeches, St. Anne's Park, Bristol 4. |
| *FISHER, A. C., M.D. Brist. .. .. .. .. .. | Roan Antelope Mine, N. Rhodesia. |
| *FITZGIBBON, G. M., M.D. Lond. .. .. .. | 1 Stoke Hill, Bristol 9. |
| *FORRESTER-BROWN, MAUD, M.D., M.S. Lond. | 22 Combe Park, Bath. |
| *FOSS, G. L., M.D., B.Ch. Cantab. .. .. .. | 1 Cambridge Park, Bristol 6. |
| *FOSS, Miss JOAN, M.B., Ch.B. Brist. .. .. | Clouds Hill House, St. George, Bristol 5. |
| *FRASER, A. D., M.A., M.B., Ch.B. Glasg. .. | 6 Rayleigh Road, Coombe Dingle, Bristol. |
| *FRASER, C. JEAN, M.B., Ch.B. Bristol .. .. | 6 Rayleigh Road, Coombe Dingle, Bristol. |
| *FRASER, D., M.B., Ch.B., Edin. .. .. .. .. .. | Westgate, Dursley, Glos. |
| *FULLER, H. L. .. .. .. .. .. .. .. .. .. | 9 Gay Street, Bath. |
| *GARDEN, R. R., M.A., M.B., B.Ch. Aberd. .. | 9 Harley Place, Clifton, Bristol 8. |
| GARLAND, E. B., M.B., C.M. Edin. .. .. .. .. | 114a Hampton Road, Redland, Bristol 6. |
| *GARMANY, G., B.Sc., M.B., Ch.B. Manch. .. | Bristol Mental Hospital, Barrow Gurney, near Bristol. |
| *GASKELL, K. H. .. .. .. .. .. .. .. .. .. | The Old House, Frenchay. |
| *GERRISH, NORMAN, M.B., Ch.B. Brist. .. .. | 2 Station Road, Keynsham, Som. |
| *GIBBS, J. R., M.B., Ch,B. Brist. .. .. .. .. | 28 Valencia Road, West Worthing, Sussex. |
| *GLASER, S., M.Sc. Cape Town, M.B.B.S., Lond. | 17 The Circus, Bath. |
| *GOLDING, H. M., *D.F.C.*, M.B., Ch.B. Brist. .. | 10 Barton Hill Road, Bristol 5. |
| *GORDON, R. G., M.D. Edin. .. .. .. .. .. | 23 Queen's Square, Bath. |
| *GORHAM, A. P., M.B., Ch.B. Brist. .. .. .. .. | 53 Westbury Road, Westbury-on-Trym. |
| *GORNALL, W. A., M.D. Brist. .. .. .. .. .. | Ulvik, Nailsea, near Bristol. |
| GRIFFITHS, CORNELIUS A. .. .. .. .. .. .. | 35 Newport Road, Cardiff. |
| *HADDEN, R., C.M. .. .. .. .. .. .. .. | 67 Charlton Road, Keynsham, Som. |
| *HAM, C. I., M.B., Ch.B. Brist. .. .. .. .. .. | 89 Stonebridge Park, Eastville, Bristol. |
| *HARRIS, H. E., M.A., M.B., B.Ch. Cantab. .. | 13 Lansdown Place, Clifton, Bristol 8. |
| *HARTLEY, GRETA, M.D. Lond. .. .. .. .. | 49 Queen's Court, Clifton, Bristol 8. |
| *HAWKINS, MONICA, M.B., Ch.B. Brist. .. .. | 7 Queen's Court, Bristol 8. |
| *HEALES, J. N., M.B., Ch.B. Brist. .. .. .. | 27 Clevedon Road, Weston-super-Mare. |
| *HECTOR, F., M.D. Lond. .. .. .. .. .. .. | 1 Victoria Square, Clifton, Bristol 8. |
| *HELLER, H. S., M.B., Ch.B., Ph.D. Camb. .. | Rodborough House, Ivywell Road, Bristol 9. |
| *HEMPHILL, R., M.D. Dub. .. .. .. .. .. | Clonora, Blackberry Hill, Fishponds. |
| *HENDERSON, JAMES, M.B., Ch.B. Aberd. .. | The City Sanatorium, Yardley Road, Birmingham. |
| *HERON, A. GORDON, M.B., Ch.B. .. .. .. .. | 237 Gloucester Road, Bristol 7. |
| *HEWER, Professor T. F., M.D. Brist. .. .. | Canynge Hall, Bristol 8. |
| *HILL, L. C., M.D. Birm. .. .. .. .. .. .. | 11 The Circus, Bath. |
| *HILL, WINIFRED, M.B., Ch.B. Brist. .. .. | c/o Lloyd's Bank, Ilford. |
| *HISCOCK, WINIFRED .. .. .. .. .. .. .. | Broken Hill, Abbots Leigh, Bristol. |
| *HOFFMAN, H. LOVELL, B.A., M.D., B.Ch. Cantab. .. .. .. .. .. .. .. .. .. | 26 The Circus, Bath. |
| *HOLLAND, L. C. .. .. .. .. .. .. .. .. .. | Upavon, Wilts. |

E

| | |
|---|---|
| *HOLLAND, W. A. L. .. .. .. .. .. .. .. | 1 Upper Belgrave Road. Clifton. |
| *HOLMES, H. W. H., B.A., M.B. Camb. .. .. | St. Aubyns, Burmham-on-Sea, Som. |
| *HOOKER, J. A., Colonel, M.B., Ch.B.Brist. .. | 16 St. Edyth's Road, Sea Mills 9. |
| *HOSKEN, J. G. F... .. .. .. .. .. .. .. | Uley, Glos. |
| HOSPITAL, BRISTOL GENERAL .. .. .. .. | |
| *HOUGHTON, LYDIA M., M.B., B.S. Lond. .. | 74 Church Road, Redfield, Bristol 5. |
| *HOWELL, T. E. .. .. .. .. .. .. .. .. | 6 Richmond Hill, Bristol 8. |
| *HUDSON, T. G. FAULKNER, M.D. Lond. .. .. | Imperial Smelting Co., Avonmouth. |
| *HUGHES, F. W. TERRELL, M.B., Ch.B. Brist. | Chew Magna, Somerset. |
| *HUNTER, F. S. .. .. .. .. .. .. .. .. | Penrose Cottage, Clifton Down, Bristol 8. |
| *HYATT, ANNIE W., M.B., B.S. Lond. .. .. | Merrymead, Shepton Mallet, Som. |
| | |
| *ILES, ANNA, M.B., Ch.B. Brist. .. .. .. | 87 Pembroke Road, Clifton, Bristol 8. |
| *ILES, A. E., *O.B.E.*, M.B., B.S. Lond. .. .. | 87 Pembroke Road, Clifton, Bristol 8. |
| INFIRMARY, BRISTOL ROYAL .. .. .. .. | |
| INGLE, C. DURBAN .. .. .. .. .. .. .. | Somerton, Somerset. |
| *ISSERLIN, B., M.B., Ch.B. Bristol .. .. .. | 21 St. John's Road, Bristol 8. |
| | |
| *JACKMAN, W. A., *T.D.*, M.B., Ch.B. Brist. .. | 15 Mortimer Road, Clifton, Bristol 8. |
| *JAMES, J. A., M.D. Lond. .. .. .. .. .. | Litfield House, Clifton Down, Bristol 8. |
| *JAMES, J. A., Senr. .. .. .. .. .. .. | Dusk, Elmlea Avenue, Stoke Bishop, Bristol, 9. |
| *JENKINS, F. G., M.B., Ch.B. Brist. .. .. .. | 51 Redcliff Hill, Bristol 1. |
| *JENKINS, P. K., *M.C.*, M.B., Ch.B. Brist. .. | St. Helena, Marine Parade, Clevedon. |
| *JOHNSON-MASON, J. .. .. .. .. .. .. | Brentry House, Brentry. |
| *JOHNSON, R. G., M.B. Lond. .. .. .. .. | 119 Redland Road, Bristol 6. |
| *JONES, D. M., M.Ch. .. .. .. .. .. .. | 18 Royal Park, Clifton, Bristol. |
| *JONES, MEGAN E. .. .. .. .. .. .. .. | Royal Infirmary, Bristol 2. |
| *JOSCELYNE, P. C., M.B., Ch.B. Brist. .. .. | 17 Falcondale Road, Westbury-on-Trym, Bristol. |
| | |
| *KEMM, N. F., M.B., B.S. Lond. .. .. .. | 9 Miles Road, Clifton, Bristol 8. |
| *KEMPTON, J. J., M.D. Brist. .. .. .. .. | Tudor House, Wokingham, Berks. |
| *KERSLEY, G. D., M.D. Cantab. .. .. .. | 6 The Circus, Bath. |
| *KINDERSLEY, C. E., M.B., Ch.B., B.A. Cantab. | 18 The Circus, Bath. |
| KING, E. F. .. .. .. .. .. .. .. .. .. | 2 Upper Wimpole Street, London, W.1. |
| *KINGSTON, S. H., M.B., Ch.B. Brist. .. .. | 3 Whiteladies Road, Clifton, Bristol 8. |
| *KYLE, H. G., M.A., M.D., B.Ch. Oxon. .. .. | 31 Westbury Road, Bristol. |
| | |
| *LACE, E. J. .. .. .. .. .. .. .. .. .. | 34 Royal York Crescent, Clifton, Bristol 8. |
| *LACE, F .. .. .. .. .. .. .. .. .. | 5 Gay Street, Bath. |
| *LACE, M. E., M.B., Ch.B. Brist. .. .. .. | 34 Royal York Crescent, Clifton, Bristol 8. |
| *LEES, E. LEONARD, M.D., C.M. Edin. .. .. | 28 Downs Park West, Bristol 6. |
| *LEGAT, P., M.B., Ch.B. Brist. .. .. .. .. | Cossham Memorial Hospital, Kingswood, Bristol. |
| LENTON, P. H., M.A., M.B., B.Chir. Cantab. | Royal Infirmary, Bristol. |
| *LEWIS, F. J., M.B., Ch.B. Brist. .. .. .. | Canynge Hall, Bristol 8. |
| *LOGAN, H. B. .. .. .. .. .. .. .. .. | Elmwood, Aberdeen Road, Cotham, Bristol 6. |
| *LOXTON, S. D., M.B., Ch.B. Brist. .. .. .. | Bristol Royal Infirmary. |
| *LUCAS, J. E. .. .. .. .. .. .. .. .. | c/o 132 York Road, Bristol 3. |
| *LUCAS, J. J. S., M.D. Lond. .. .. .. .. | 186 Wells Road, Knowle, Bristol 4. |
| *LUCAS, J. V., M.A., M.B., B.Ch. Cantab. .. | 186 Wells Road, Knowle, Bristol 4. |
| *LUCAS, R. P., M.B., Ch.B. Brist. .. .. .. | Northways, Fallodon Way, Henleaze. |
| | |
| *MACDONALD, G., M.B., Ch.B. .. .. .. .. | 61 Don Street, Invercargill, N.Z. |
| *MCKENZIE, W. G., *M.C.* .. .. .. .. .. | 11 Bath Road, Reading. |
| *MACLACHLAN, A. M., M.B., Ch.B. Glasg. .. | 65 Redcliff Hill, Bristol 1. |
| *MACLAREN, CATHERINE J., M.B., Ch.B. Edin. | Hereford House, Clifton Down. |
| MACLEAN, SIR EWEN J., M.D., C.M. Edin. .. | 12 Park Place, Cardiff |
| *MACLEOD, G., M.A. Glas., M.D., .. .. .. | Wargrave, Clevedon, Somerset. |
| *MARSHALL, A. H., M.B., Ch.B. Brist. .. .. | 281 Canford Lane, Bristol. |

| | |
|---|---|
| MARSHALL, A. L., M.B., B.A. Cantab. .. .. | Laxfield, Woodbridge, Suffolk. |
| *MARTIN, P. S., *M.C.* .. .. .. .. .. .. .. | Victoria Quadrant, Weston-super-Mare. |
| *MARWOOD, S. F., M.D., B.S. Lond. .. .. .. .. | Troy House, Kingswood, Bristol. |
| *MASON, J., M.B., Ch.B. Edin. .. .. .. .. .. | 7 Westbury Road, Westbury-on-Trym, Bristol. |
| *MAWSON, EVELYN, M.D. Liverp. .. .. .. .. | Frenchay Park Sanatorium, Bristol. |
| MIALL, C. L'O .. .. .. .. .. .. .. .. .. | Elm Hayes, Paulton, near Bristol. |
| *MILLING, T., M.B., Ch.B. Belf. .. .. .. .. .. | 1 Vicarage Road, Southville, Bristol 3. |
| *MOHAN, S. L., M.B., B.S. Punjab. .. .. .. .. | 255 Stapleton Road, Bristol 5. |
| *MOORE, C. A., M.B., M.S. Lond. .. .. .. .. | South Lodge, Kingsweston Road, Henbury, Bristol. |
| *MOORE, R. H., M.B., Ch.B. Brist. .. .. .. .. | White Cross Court, Wells Road, Bristol 4. |
| *MORGANS, C. C., M.A., M.B., B.Ch. Cantab. .. | 65 Lower Redland Road, Bristol 6. |
| *MUNDY, G. S., M.B., B.Ch. Brist. .. .. .. .. | The Knoll, Chipping Sodbury, Glos. |
| *MURPHY, Col. D. F., *M.C.*, M.B., N.U.I., I.M.S. (Retd.) .. .. .. .. .. .. .. .. .. | 25 St. Martin's Road, Bristol 4. |
| *MURPHY, F. D., *O.B.E.*, B.Sc., M.B., B.Ch., B.A.O., N.U.I. .. .. .. .. .. .. .. | 20 Gay Street, Bath. |
| *MUSSEN, R. W., M.D. Belfast .. .. .. .. .. | Royal Naval Medical School, Clevedon. |
| *MYLES, Q. St. L., M.A., B.M., B.Ch. Oxon. .. | 7 Apsley Road, Clifton, Bristol 8. |
| | |
| *NICHOLSON-LAILEY, J. R. .. .. .. .. .. .. | Elmfield House, South Road, Taunton. |
| *NICHOLSON, M. A., M.B., Ch.B. Brist. .. .. | 2 Elmlea Avenue, Stoke Bishop, Bristol. |
| *NIXON, DOREEN, M.B., B.S. Lond. .. .. .. .. | 7 Lansdown Place, Clifton, Bristol 8. |
| *NIXON, J. A., *C.M.G.*, M.D. Cantab. .. .. | 7 Lansdown Place, Clifton, Bristol 8. |
| *NOBLE, J. I., M.B., Ch.B. Liverp. .. .. .. .. | 20 Richmond Hill, Clifton, Bristol 8. |
| *NORMAN, R. M., M.D. Brist. .. .. .. .. | Highwood, Stapleton Park, Bristol. |
| *NORRIS, C. T. .. .. .. .. .. .. .. .. .. | 11 Bryant's Hill, Bristol 5. |
| | |
| *O'DONOHOE, MONICA A., M.B., B.Ch., N.U.I. | 1 Beaconsfield Road, Bristol 8. |
| *OLIFF, D. E., M.B., Ch.B. .. .. .. .. .. .. | 8 Lansdown Crescent, Bath. |
| *ORMEROD, G. L., M.A., M.B., B.Ch. Cantab .. | 2 Henleaze Road, Westbury-on-Trym, Bristol. |
| *ORR-EWING, H. J., *M.C.*, M.D. Lond. .. .. | 1 Beaufort Buildings, Clifton, Bristol 8. |
| *ORTON, B. F., M.B., Ch.B. Brist. .. .. .. .. | 30 Cornwallis Crescent, Clifton, Bristol 8. |
| *ORTON, R. W., M.B., Ch.B. Brist. .. .. .. .. | 30 Cornwallis Crescent, Clifton, Bristol 8. |
| | |
| *PALIN, A. G., B.M., B.Ch. Oxon. .. .. .. .. | Litfield House, Clifton Down, Bristol 8. |
| *PARKES, J. A., M.B., Ch.B. Liverp. .. .. .. | 8 South Road, Redland, Bristol 6. |
| *PARKES, P. W. J. .. .. .. .. .. .. .. .. | 164 Filton Avenue, Bristol 7. |
| *PARRY, R. H., M.B., B.S.Lond. .. .. .. .. | Public Health Offices, Bristol 1. |
| PARSONS, Sir J. H., M.B., B.S., B.Sc. Lond... | 54 Queen Anne Street, London, W. |
| *PAUL, R. G. .. .. .. .. .. .. .. .. .. | 58 St. John's Road, Bristol 8. |
| *PAULI, C. H. .. .. .. .. .. .. .. .. .. | Pagan's Hill Farm, Chew Stoke. |
| *PEACH, A. N. H., M.B., Ch.B. Brist. .. .. | Bristol Royal Infirmary. |
| *PEARSON, G. M. .. .. .. .. .. .. .. .. | West Harptree, near Bristol. |
| *PERKS, RUTH, M.B., Ch.B. Brist. .. .. .. .. | Auckland House, Clifton Down. |
| *PERRY, B., M.D., Ch.B. Brist. .. .. .. .. .. | Royal Infirmary, Bristol. |
| *PETTY, W. J., M.B., B.S. Lond. .. .. .. .. | "Craigside," Worle, Weston-super-Mare. |
| PHELPS, P. C., M.B., Ch.B. Brist. .. .. .. | 23 St. Alban's Road, Bristol 6. |
| *PHILLIPS, P., M.B., Ch.B. Brist. .. .. .. .. | Southmead Hospital, Bristol. |
| *PIRRIE, A. L., M.B., Ch.B. Glasg. .. .. | 528 Kensington Hill, Bristol. |
| *POCOCK, J. A., M.A., M.B., B.Ch. Cantab. .. | Medical Postgraduate Dept., The University, Bristol. |
| *POND, MRS. M. H., M.B., B.Ch. Cantab. .. | 24 Cherrington Road, Henleaze, Bristol 7 |
| *PORTER CHARLES, M.A. Oxon. .. .. .. .. .. | 124a Redland Road, Bristol 6. |
| PORTER, C. P. .. .. .. .. .. .. .. .. .. | 27 Church Street, Kidderminster. |
| *POTTER, MABEL F., M.D., Brist. .. .. .. .. | 19 Royal Park, Clifton, Bristol 8. |
| *POWELL, H. F., M.D. Brux. .. .. .. .. .. | Ellenborough Park, Weston-super-Mare. |
| *PRATT, W. W. .. .. .. .. .. .. .. .. .. | Feltham Farm, Pucklechurch. |
| *PRICE, ANNIE B., B.Sc., M.B., Ch.B. Glasg. .. | Castleton Nursing Home, Bolters Lane, Banstead, Surrey. |
| *PRICE, C. H. G., M.B., Ch.B. Brist. .. .. | Claverham House, Claverham, Yatton. |

| | |
|---|---|
| *PRIDIE, K. H., M.B., B.S. Lond. .. .. .. .. | 58 St. John's Road, Clifton, Bristol 8. |
| *PRIDIE, MRS. KENNETH .. .. .. .. .. .. .. | 58 St. John's Road, Bristol 8. |
| *PROWSE, D. C., M.B., Ch.B. .. .. .. .. .. | Wigmore House, Thornbury, Near Bristol. |
| *PYKE, H. D., M.B., Ch.B. Brist. .. .. .. .. | 88 Redland Road, Bristol 6. |
| *RANKIN, P. C., M.B., Ch.B. Glasg. .. .. .. .. | Lawrence Hill House, Bristol. |
| *REID, A. C., B.Sc., M.B., Ch.B. Edin. .. .. | Olveston, near Bristol. |
| *RICHARDS, F. B., M.B., Ch.B. Brist. .. .. | 23 Grange Park, Westbury-on-Trym, Bristol. |
| *RIDGWAY, J., M.B., Ch.B. Brist. .. .. .. .. | Hanham, Bristol. |
| *ROBERTS, J. A. F., M.A. Cantab., M.B., Ch.B., D.Sc. Edin. | Stoke Park Colony, Bristol. |
| *ROBERTS, J. A. L., *M.C.*, Ch.B. Brist. .. .. | 75 High Street, Kingswood, Bristol. |
| *ROBERTSON, D., M.B., Ch.B. Edin. .. .. .. .. | 2 Knowle Road, Knowle, Bristol 4. |
| *ROBERTSON, L. G., *M.C.*, M.B., Ch.B. Brist. .. | 162 Redland Road, Bristol 6. |
| *ROGERS, H., M.D. Brist. .. .. .. .. .. .. | "Marguerite," Catlein's Lane, Eastcote, Middlesex. |
| *ROLFE, R., B.A., M.B., B.C. Cantab. .. .. | Park House, St. Andrew's Road, Avonmouth. |
| *ROSS, A. I., M.D. .. .. .. .. .. .. .. .. | 18 Grange Park, Westbury-on-Trym, Bristol. |
| *ROWLANDS, R. D. .. .. .. .. .. .. .. .. | Elmfield House, South Road, Taunton. |
| *ROWLEY, A. T. .. .. .. .. .. .. .. .. .. | 13 Walliscote Road, Weston-super-Mare. |
| *RUDOLF, G. DE M. .. .. .. .. .. .. .. .. | Litfield House, Clifton, Bristol 8 |
| *RYAN, P. B., M.B., Ch.B. Brist. .. .. .. | The Paddock, Frenchay. |
| *SAMPSON, O. E. L., M.B., Ch.B. Brist. .. .. | 77 Stackpool Road, Bristol 3. |
| *SANSOM, J. R. E., B.Sc., M.B., Ch.B. Brist. .. | 149 Bell Hill Road, St. George, Bristol 5. |
| *SCARFF, G., M.B., Ch.B. Edin. .. .. .. .. .. | 83 Pembroke Road, Clifton, Bristol 8. |
| *SHEPHERD, H. L., M.B., Ch.M. Brist. .. .. | 79 Pembroke Road, Clifton, Bristol 8. |
| *SHORE, T. L. H., M.A., M.B., B.Ch. Cantab. .. | Linden Lodge, Haines Hill, Taunton. |
| *SHORT, A. R., M.D. Lond. .. .. .. .. .. .. | 69 Pembroke Road, Clifton, Bristol 8. |
| *SHORT, E. S., M.B., Ch.B. Brist. .. .. .. | Winsley Sanatorium, near Bath. |
| *SILVEY, STEWART, M.B., Ch.B. Brist. .. .. | "Denewood," Coombe Dingle, Bristol 9. |
| *SIMPSON, T. E. N. .. .. .. .. .. .. .. .. | "West Hythe," Old Patchway, Bristol. |
| *SMART, G. A., M.D. Durh. .. .. .. .. .. | Bristol Royal Infirmary. |
| *SMYTHE, H. J. D., *M.C.*, M.D., M.S. Lond. .. | Southmead Hospital, Bristol. |
| *SNAWDON, J. W. E., M.B., Ch.B., B.D.S. Brist. | Litfield House, Clifton, Bristol 8. |
| *SOLTAU, DAVID K., M.A., M.B., B.Ch. Cantab. | 19 The Avenue, Clifton, Bristol 8. |
| *SOMERS, MARY A. E. .. .. .. .. .. .. .. | 2 Ashcombe Road, Weston-super-Mare. |
| *SPARKS, J. V., B.A. Cantab. .. .. .. .. .. | The Middle House, Felton, Som. |
| *SPOOR, A., M.B., Ch.B. Cantab. .. .. .. .. | The Hydro, Bristol 1. |
| *STEINER, G., M.D. Vienna .. .. .. .. .. | 23 Manilla Road, Clifton, Bristol 8. |
| *STEPHEN, R. J. .. .. .. .. .. .. .. .. .. | 92 Redland Road, Bristol 6. |
| *STROVER, H. W. M., *O.B.E.*, M.B., Ch.B. Aberd. | 90 Redland Road, Bristol 6. |
| *STRUTHERS, A. J., M.B., Ch.B. Glasg. .. .. | 100 Church Road, Redfield, Bristol 5. |
| *STRUTHERS, GEO., M.B., Ch.B. Glasgow .. .. | 100 Church Road, Redfield, Bristol 5. |
| Students Common Room, Bristol Royal Infirmary | |
| *SULLIVAN, H. W., M.B., Ch.B. .. .. .. .. .. | Stoke Park Colony, Stapleton, Bristol 5. |
| *SUTTON, G. E. F., M.D. Lond. .. .. .. .. | 1 Pembroke Road, Clifton, Bristol 8. |
| *TALLACK, MAJOR R. J. K., R.A.M.C. .. .. | Medical Officer, c/o Postmaster, Zanzibar. |
| *TASKER, D. G. C., M.B., M.S. Lond. .. .. | 9 College Fields, Clifton, Bristol 8. |
| *TAYLOR, A. L., M.D. Lond. .. .. .. .. .. | The General Hospital, Bristol 1. |
| TAYLOR, A. L. .. .. .. .. .. .. .. .. .. | 35b Apsley Road, Bristol 8. |
| *TAYLOR, MARY, M.B., B.S. .. .. .. .. .. | Burden Neurological Institute, Fishponds, Bristol. |
| THIN, J. .. .. .. .. .. .. .. .. .. .. | 54 and 55 South Bridge, Edinburgh. |
| *TODD, A. T., *O.B.E.*, M.D. Edin. .. .. .. .. | Royal Park House, Clifton, Bristol 8. |
| *TOMLINSON, STERLING, M.B., B.Ch., B.A.O. Dub. | 41 Newmarket Road, Norwich. |
| *TRIBE, WINIFRED N., M.B., Ch.B. Brist. .. | c/o Ebenezer Congregational Church, 10 Main Street, Ferrierastown, Johannesburg. |
| TRINICK, RICHARD H. .. .. .. .. .. .. .. | 42 Cherry Valley Gardens, Belfast. |
| TRIPP, DOROTHY M. H. .. .. .. .. .. .. .. | Mission Hospital, Karim Nagar, Nizam's Dominions, South India. |
| *TRYON, VICTORIA S., M.B., Ch.B. Brist. .. | 3 Harley Place, Clifton, Bristol 8 |

| | |
|---|---|
| WAKE, S. C., M.B., Ch.B. Brist. .. .. .. | "Stanhope," Bideford, N. Devon. |
| *WALKER, CYRIL H., M.B. Cantab. .. .. .. | Harewood, Clapton-in-Gordano, Som. |
| *WALKER, Prof. MILNES, M.S. Lond. .. .. | Wrington, Bristol. |
| *WANSBROUGH, T. B., M.B., Ch.B. Brist. .. | 23 Pembroke Road, Clifton, Bristol 8. |
| *WARREN, R., M.A., M.D. Oxon. .. .. .. | Heathgates, Weston-super-Mare. |
| *WATSON-WILLIAMS, E., *M.C.*, M.B. Cantab., Ch.M. Brist. | 65 Pembroke Road, Clifton, Bristol 8. |
| WEDDELL, C. W. .. .. .. .. .. .. .. .. .. | Boydville, 46 Miller Street, West Melbourne, Australia. |
| *WEEKS, A. .. .. .. .. .. .. .. .. .. .. | 39 Badminton Road, Downend. |
| *WEST, H. F., M.B., B.S. Lond. .. .. .. | 11 Eaton Crescent, Clifton, Bristol 8. |
| *WESTHEAD, PAMELA, M.B., Ch.B. .. .. .. | Royal Infirmary, Bristol. |
| *WETHERED, R. R., B.A., B.M., B.Ch. Oxon. .. | Crossways, Stoke Lane, Westbury-on-Tryn, Bristol. |
| *WHITE, R. F., M.B., Ch.B. Brist. .. .. .. | "Wellclose," High Street, Nailsea. |
| *WILBOND, R. G. .. .. .. .. .. .. .. .. .. | 76 Chesterfield Road, St. Andrew's Park, Bristol. |
| *WILLIAMS, I., M.B., Ch.B. Brist. .. .. .. | "Ardrem," Hill View, Henleaze, Bristol. |
| *WILLIAMS, S. R., M.B. Lond. .. .. .. .. | Buckfastleigh, Devon. |
| *WILLIAMSON, Col. M. J., *M.C.*, M.B., Ch.B. Aberd. | Rodney House, Clifton, Bristol 8. |
| *WILLOUGHBY, J., M.B., B.Ch., B.A.O. Dub. .. | "Kingsholme," Clevedon Road, Nailsea. |
| *WILSON, W. HARGRAVE .. .. .. .. .. .. | P.O. Box 681, Nairobi, Kenya. |
| *WOOD, D. .. .. .. .. .. .. .. .. .. .. | 105 Pembroke Road, Clifton, Bristol 8. |
| *WOOD, W. V., *M.C.*, B.A. Cantab. .. .. .. | Henley Lodge, Yatton, Som. |
| *WOODMAN, DOROTHY, M.D. Lond. .. .. .. | 10 Tyndall's Park Road, Clifton, Bristol 8. |
| *WOOLLEY, W., M.B., Ch.B. Brist. .. .. .. | 239 Cranbrook Road, Redland, Bristol 6. |
| *WOOLMER, RONALD, B.A., B.M., B.Ch. Oxon. | 21 Downleaze, Stoke Bishop, Bristol 9. |
| *WRIGHT, A. J. M., M.B., B.S. Lond. .. .. | Harley Cottage, Clifton Park, Clifton, Bristol 8. |
| *WRIGHT, WINIFRED M., M.B., B.S. Lond. .. | Merrymead, Shepton Mallet, Som. |
| *ZEALAND, L., M.D. Man. .. .. .. .. .. .. | Brecon Lodge, Westbury-on-Trym, Bristol. |

